# Polymorphisms of *IFN* signaling genes and *FOXP4* influence the severity of COVID-19

**DOI:** 10.1186/s12879-024-09040-6

**Published:** 2024-03-01

**Authors:** Feng Zhang, Pingping Zhou, Liangliang Wang, Xinzhong Liao, Xuejie Liu, Changwen Ke, Simin Wen, Yuelong Shu

**Affiliations:** 1 School of Public Health (Shenzhen), Sun Yat-Sen University, Shenzhen, 518107 P. R. China; 2 Guangdong Provincial Institute of Public Health, Guangdong Provincial Center for Disease Control and Prevention, Guangzhou, P. R. China; 3 Guangdong Provincial Center for Disease Control and Prevention, Guangzhou, P. R. China; 4 Guangzhou First People’s Hospital, the Second Affiliated Hospital of South China University of Technology, Guangzhou, P. R. China; 5 Key Laboratory of Pathogen Infection Prevention and Control (MOE), State Key Laboratory of Respiratory Health and Multimorbidity, National Institute of Pathogen Biology, Chinese Academy of Medical Sciences & Peking Union Medical College, Beijing, 102629 P. R. China

**Keywords:** MX1, FOXP4, Single nucleotide polymorphisms, COVID-19

## Abstract

**Background:**

The clinical manifestations of COVID-19 range from asymptomatic, mild to moderate, severe, and critical disease. Host genetic variants were recognized to affect the disease severity. However, the genetic landscape differs among various populations. Therefore, we explored the variants associated with COVID-19 severity in the Guangdong population.

**Methods:**

A total of 314 subjects were selected, of which the severe and critical COVID-19 patients were defined as “cases”, and the mild and moderate patients were defined as “control”. Twenty-two variants in interferon-related genes and *FOXP4* were genotyped using the MassARRAY technology platform.

**Results:**

*IFN* signaling gene *MX1* rs17000900 CA + AA genotype was correlated with a reduced risk of severe COVID-19 in males (*P* = 0.001, OR = 0.050, 95%CI = 0.008–0.316). The AT haplotype comprised of *MX1* rs17000900 and rs2071430 was more likely to protect against COVID-19 severity (*P* = 6.3E-03). *FOXP4* rs1886814 CC genotype (*P* = 0.001, OR = 3.747, 95%CI = 1.746–8.043) and rs2894439 GA + AA genotype (*P* = 0.001, OR = 5.703, 95% CI = 2.045–15.903) were correlated with increased risk of severe COVID-19. Haplotype CA comprised of rs1886814 and rs2894439 was found to be correlated with adverse outcomes (*P* = 7.0E-04). *FOXP4* rs1886814 CC (*P* = 0.0004) and rs2894439 GA + AA carriers had higher neutralizing antibody titers (*P* = 0.0018). The CA + AA genotype of MX1 rs17000900 tended to be correlated with lower neutralizing antibody titers than CC genotype (*P* = 0.0663), but the difference was not statistically significant.

**Conclusion:**

Our study found a possible association between *MX1* and *FOXP4* polymorphisms and the severity of COVID-19. Distinguishing high-risk patients who develop severe COVID-19 will provide clues for early intervention and individual treatment strategies.

**Supplementary Information:**

The online version contains supplementary material available at 10.1186/s12879-024-09040-6.

## Introduction

The pandemic of coronavirus disease 2019 (COVID-19), caused by severe acute respiratory syndrome coronavirus 2 (SARS-CoV-2), has infected billions of people worldwide and posed an enormous threat to global public health and economies. The clinical manifestations of SARS-CoV-2 infection appear widely, ranging from asymptomatic infection, and mild upper respiratory tract illness to severe viral pneumonia with respiratory failure and even death [1]. Reported risk factors for severe COVID-19 include male sex [2], older age, and some comorbidities, such as chronic lung disease, cardiovascular disease, hypertension, diabetes, and obesity [3].

Additionally, host genetic variants have also been shown to modulate the risk of infection and disease severity. Large-scale genome-wide association studies (GWASs) in populations of European ancestry identified some genomic loci associated with COVID-19 severity and susceptibility [4–6], including rs11385942 (*LZTFL1*), rs1886814 (near *FOXP4*), rs657152 (*ABO*), rs10735079 and rs10774671 (*OAS* gene cluster), rs74956615 (near *TYK2*), rs2109069 (*DPP9*), and rs2236757 (*IFNAR2*). Several genetic loci related to critical illness in COVID-19 belonged to interferon (IFN) signaling, which was further supported by a recent study that several loss-of-function variants in the IFN pathway were enriched in severe COVID-19 patients [7].

The interferon response functions as the major first line of defense against viruses, including SARS-CoV-2. IFNs interact with their receptors to activate downstream signaling cascades that eventually induce numerous IFN-stimulated genes with various antiviral activities, which contributes to effectively establishing an antiviral state in infected and surrounding cells [8]. Genetic variants in IFN-related genes can influence host antiviral response by affecting IFN production and serum levels. In addition, genetic variants in FOXP4 were associated with severe COVID-19 based on European. Of note, the allele and genotype frequencies of these single nucleotide polymorphisms (SNPs) vary among different populations due to the human genetic background. Recently, most of the associated variants biased towards European-ancestry samples and thus might not be generalizable to other non-European populations, especially for *LZTFL1* rs11385942 [4] and *TYK2* rs74956615 [6], which were monomorphic in the Asian population. To better understand the host mechanisms that lead to severe COVID-19 among the Guangdong population, we investigated some of the major candidate variants that have been identified as potential genetic factors based on European populations or meta-analyses with multiple populations, as well as common variants of IFN signaling genes that have been reported to be related to the severity of other viral infectious diseases.

Therefore, we selected twenty-two SNPs of *IFN* signaling genes and *FOXP4*. Our study aimed to investigate the association of *IFN* signaling genes and *FOXP4* polymorphisms with the severity of COVID-19 in Guangdong population, and thus provide information for effective prevention and individual treatment strategies in the future.

## Materials and methods

### Study participants and samples

﻿The subjects were excluded from the enrollment if they were not infected with SARS-CoV-2, or their blood sample was inadequate for genotyping or serological tests. 314 COVID-19 cases were included in this study from 23 January to 1 March 2020 in Guangdong, China. The clinical characteristics and outcomes of all subjects were retrieved from the Guangdong Provincial COVID-19 surveillance network database. Blood samples were collected at a median of 31 days post-illness onset by the Guangdong Provincial Center for Disease Control and Prevention, and serum samples were isolated immediately and stored at − 80 °C. Both blood clots and serum samples were heat-inactivated at 56 °C for 30 30 min before tests. Clinical classifications of COVID-19 patients (mild, moderate, severe, or critical) were made according to the Diagnosis and Treatment Protocol for COVID-19 (version 7.0) [9]. There were 64 severe or critical COVID-19 patients (designated as cases) and 250 mild or moderate patients (designated as controls), of which only 61 cases and 231 controls included comorbidities data.

All the work was approved by the biomedical research ethics committee, the public health school (Shenzhen) of Sun Yat-sen University.

### Genomic DNA preparation

The genomic DNA was extracted using the TIANamp Blood Clot DNA Kit (TIANGEN, DP335) according to the manufacturer’s protocol. DNA purity was evaluated by OD260/OD280 and OD260/OD230 ratios (NanoDrop, Thermo Fisher Scientific, USA). The acceptance criteria for DNA purity were OD260/OD280 ratios of 1.7–2.0 and OD260/OD230 ratios > 1.0. DNA degradation was assessed on a 1% agarose gel using an appropriate size standard control and showed a single bright band.

### SNP selection and genotyping

We summarized the genomic loci related to severe COVID-19 reported in the previous GWASs or related to the severity of other viral infectious diseases, and twenty-two SNPs in *IFN* signaling genes and *FOXP4* were selected*.* The minimum allele frequencies (MAFs) of all SNPs were greater than 0.05 in the Chinese population according to the SNP database in NCBI (https://www.ncbi.nlm.nih.gov/snp/). These SNPs of *IFN* signaling genes including: TLR3 rs3775291 and rs5743313, TLR7 rs3853839, DDX58 rs3739674 and rs10813831, IFIH1 rs1990760 and rs2111485, IFNAR2 rs2236757, rs13050728, rs1051393 and rs2229209, TYK2 2304256, MX1 rs17000900 and rs2071430, OAS1 rs10774671, rs1131454, and rs2660, OAS3 rs10735079, rs2285933 and rs1859330. Moreover, rs1886814 and 2894439 in the FOXP4 locus were selected, significantly associated with severe COVID-19 in European populations, and meta-analyses with multiple populations. Basic information regarding the SNPs was demonstrated in Supplementary Table 1.

Genotyping of each SNP was performed using the MassARRAY technology platform (Sequenom, San Diego, California, USA) and determined by BioMiao Biological Technology (Beijing, China).

### Genetic models

In this study, four genetic models were applied for the analysis. The codominant genetic model was used to compare the frequencies of the three genotypes. The overdominant genetic model compared the frequencies of homozygotes with heterozygotes to analyze the homozygous effect. The dominant genetic model compared the frequencies of wild-type homozygotes with other phenotypes. The recessive genetic model compared the frequencies of mutant homozygotes with other phenotypes. According to the results of the data analysis, we selected the optimum genetic model with the smallest *P* values.

### Microneutralization assay

According to standard neutralization test protocols, microneutralization antibody assays for SARS-CoV-2 were performed in a BSL-3 laboratory. Briefly, serum samples were inactivated at 56 °C for 30 min before use, and then serially diluted in 2-folds with minimum essential medium from 1:4 to 1:1024. Diluted sera were mixed with 100 TCID_50_ of SARS-CoV-2 (GISAID accession ID. EPI_ISL_403934), and incubated at 37℃ for 2 h (triplicate repetition). Thereafter, the mixture was added to Vero-E6 cells and incubated at 37℃ with 5% CO_2_. Viral-induced cytopathic effect was monitored daily for seven days. Cell, serum, and virus controls were included in each plate. Virus back titration was conducted in each test. The microneutralization antibody titer was recorded as the highest dilution with 50% inhibition of the cytopathic effect.

### Statistical analysis

The Hardy–Weinberg equilibrium (HWE) test was conducted to assess the genotype frequencies of SNPs among subjects. Assessment of the linkage disequilibrium (LD) and haplotype analysis was performed by SNPStats (https://www.snpstats.net/). Continuous variables with normal distribution through the Kolmogorov–Smirnov test were described as the mean ± SD and analyzed by One-way ANOVA. Continuous variables without normal distribution were described as the medians (interquartile ranges, IQRs) and analyzed by Mann–Whitney U tests. Categorical data were summarized as frequencies (percentages) and compared using the χ^2^ test or Fisher's exact probability test between the cases and controls, depending on the sample sizes. Associations between SNPs and the severity of COVID-19 were calculated using univariable and multivariable logistic regression models adjusted for gender, age and comorbidities (including hypertension, diabetes, and cardiovascular diseases) through different genetic models. ﻿Associations between significant SNPs and neutralizing antibody levels were calculated using linear regression analysis. The level of statistical significance was *P* < 0.05 with two-tailed. The significance level was turned to* P* < 2.50E-03 (0.05/(22–2) = 2.50E-03) according to Bonferroni correction when analyzing the relationship between SNPs and the severity of COVID-19. The minimum sample size was estimated using Quanto software (v1.2.4). All analyses were performed by the SPSS software (V.26.0) or GraphPad Prism(v9).

## Results

### Characteristics of subjects

A total of 314 subjects were included in this study. As shown in Table 1, there were 153 men (48.7%) and 161 women (51.3%). The median age of the 314 patients was 45.0 years (IQR 33.0–59.3), and the severe or critical patients (62.0(56.0–66.5)) older than mild or moderate patients (40.0(32.0–55.0)) (*P* = 5.11E-15). We performed the χ2 test and statistically proved that age and male were risk factors for severe symptoms of COVID-19 (*P* = 6.89E-14 and *P* = 0.002, respectively). The most common comorbidities were hypertension (*N* = 23, 37.7%), diabetes (*N* = 15, 24.6%), and cardiovascular diseases (*N* = 13, 21.3%) among severe and critical patients. After adjusting for age and gender, we found that the presence of comorbidities would affect the patients’ severity, and at least one comorbidity was more likely to develop severe COVID-19 (*P* = 6.00E-05). As previous studies reported [3], comorbidities played an important role in the poor outcomes of COVID-19 patients.

**Table 1:** Clinical characteristics of the COVID-19 patients in this study

Categories	Case (*n* = 64)	Control (*n* = 250)	Total (*n* = 314)	*P*
Age, n(%)				6.89E-14
< 60	25(39.1)	211(84.4)	236(75.2)	
≥ 60	39(60.9)	39(15.6)	78(24.8)	
Median(IQR)	62.0(56.0–66.5)	40.0(32.0–55.0)	45.0(33.0–59.3)	5.11E-15^a^
Gender, n(%)				0.002
Male	42(65.6)	111(44.4)	153(48.7)	
Female	22(34.4)	139(55.6)	161(51.3)	
Comorbidities,n(%)^b^				
None	23(37.7)	186(80.5)	209(71.6)	6.00E-05^c^
Hypertension	23(37.7)	18(7.8)	41(14.0)	0.002^c^
Diabetes	15(24.6)	13(5.6)	28(9.6)	0.003^c^
Cardiovascular diseases	13(21.3)	5(2.2)	18(6.2)	0.023^c^
Liver disease	2(3.3)	5(2.2)	7(2.4)	0.792^c^
Lung disease	4(6.6)	5(2.2)	9(3.1)	0.348^c^
Chronic renal diseases	6(9.8)	1(0.4)	7(2.4)	0.159^c^
Cancers	1(1.6)	2(0.9)	3(1.0)	0.685^c^

^a^Mann-Whitney U test

^b^61 cases and 231 controls only

^c^Multivariable logistic regression analysis adjusted for age and gender

### Association of 22 candidate genetic variants with the severity of COVID-19

Two of the 22 selected SNPs evaluated did not meet the Hardy–Weinberg equilibrium (rs13050728 in IFNAR2, rs3853839 in TLR7) (*P* < 0.001) (Supplementary Table 1) and thus were not included in the subsequent analyses. The univariable analysis indicated that two SNPs including *FOXP4* rs1886814 and rs2894439, had different genotype or allele frequencies between the cases and controls. The frequencies of rs1886814 CC genotype and C allele were higher in severe or critical patients than in mild or moderate patients (*P* = 2.59E-04 and* P* = 2.33E-04, respectively), and rs2894439 AA genotype and A allele had the same tendency (*P* = 0.001 and *P* = 2.33E-04, respectively), which were still significant after the Bonferroni correction (*P* < 2.50E-03) (Table 2). The frequencies of *MX1* rs17000900 A (*P* = 0.027) allele in the case group tended to be lower than that in the control group, but the difference was not significant after the Bonferroni correction (Table 2). For the remaining 17 SNPs, there was no significant association between SNPs and the severity of COVID-19 in our study, as showed in Supplementary Table 2.

**Table 2:** Genotype and allele distributions of candidate SNPs between the two groups (significance only)

SNP	Genotype/Allele	Case (*n* = 64)	Control (*n* = 250)	*P*^a^	OR(95%CI)^b^	*P*^b^
*MX1* rs17000900	CC	54(84.4)	180(72.0)	0.089	1.000	
	CA	10(15.6)	63(25.2)		0.529(0.254–1.101)	0.089
	AA	0(0.0)	7(2.8)		-	-
	C	118(92.2)	423(84.6)	0.027		
	A	10(7.8)	77(15.4)			
*FOXP4* rs1886814	AA	13(20.3)	90(36.0)	3.89E-04	1.000	
	AC	29(45.3)	125(50.0)		1.606(0.791–3.261)	0.190
	CC	22(34.4)	35(14.0)		4.352(1.977–9.578)	2.59E-04
	A	55(43.0)	305(61.0)	2.33E-04		
	C	73(57.0)	195(39.0)			
*FOXP4* rs2894439	GG	10(15.6)	96(38.4)	0.001	1.000	
	GA	35(54.7)	113(45.2)		2.973(1.400–6.317)	0.005
	AA	19(29.7)	41(16.4)		4.449(1.904–10.393)	0.001
	G	55(43.0)	305(61.0)	2.33E-04		
	A	73(57.0)	195(39.0)			

*SNP* single nucleotide polymorphism, *OR* odds ratio, *CI* confidence interval

^a^The *P* values were calculated by the Chi-square test

^b^ORs and *P* values were calculated by univariable logistic regression analysis

The multivariable logistic regression analysis adjusted for gender, age, and comorbidities was performed for variants with *P* < 0.01 in univariable analyses. Multivariable logistic analysis results of all 20 variants using the optimum genetic model were shown in Supplementary Table 3. Compared with the AA + AC genotype of rs1886814, the CC genotype was associated with a higher risk of developing severe COVID-19 (*P* = 0.001, OR = 3.747, 95% CI = 1.746–8.043). Similarly, subjects with the GA + AA genotype of rs2894439 were more likely to suffer severe COVID-19 than subjects with the GG genotype (*P* = 0.001, OR = 5.703, 95% CI = 2.045–15.903) (Table 3). There were still significant differences after the Bonferroni correction.

**Table 3:** Multivariable logistic regression analysis adjusted for age, gender and comorbidities

SNP	Genetic model	Genotype	Case	Control	*P*	OR(95%CI)
*FOXP4* rs1886814	Recessive	AA + AC	39(63.9)	200(86.6)		1.000
		CC	**22(36.1)**	**31(13.4)**	0.001	3.747(1.746–8.043)
*FOXP4 *rs2894439	Dominant	GG	7(11.5)	88(38.1)		1.000
		GA + AA	**54(88.5)**	**143(61.9)**	0.001	5.703(2.045–15.903)

ORs and *P* values were calculated by multivariable logistic regression analysis adjusted for age, gender and comorbidities

### Gender stratification analysis

After the Bonferroni correction, the stratified analysis found that *MX1* rs17000900 CA + AA genotype was related to a reduced risk of severe COVID-19 compared to the CC genotype in males (*P* = 0.001, OR = 0.050, 95%CI = 0.008–0.316), but it was not significant in females (Table 4 and Supplementary Table 4).

**Table 4:** Multivariable logistic regression analysis of candidate variants in different gender groups

SNP	Genetic Model	Genotype	Male				Female			
			Case	Control	*P*	OR(95%CI)	Case	Control	*P*	OR(95%CI)
*MX1* rs17000900	Dominant	CC	37(92.5)	70(68.0)		1.000	15(71.4)	95(74.2)		1.000
		CA + AA	3(7.5)	33(32.0)	0.001	0.050(0.008–0.316)	6(28.6)	33(25.8)	0.588	1.391(0.422–4.592)

ORs and *P* values were calculated by multivariable logistic regression analysis adjusted for age and comorbidities

### Association of SNPs with neutralizing antibody titers

**Fig. 1:**
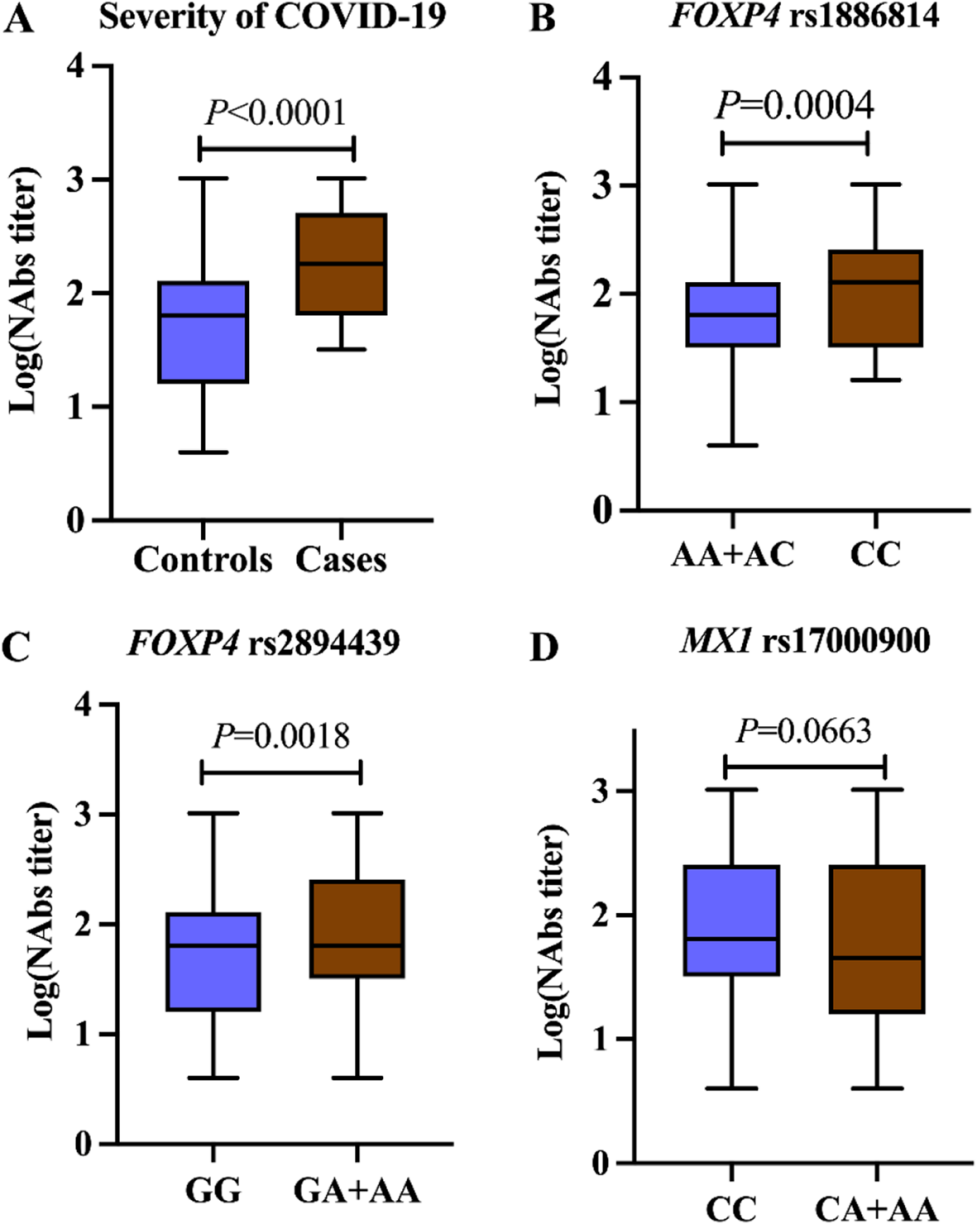
Comparison of neutralizing antibody titers among COVID-19 patients. **A** The comparison of neutralizing antibody titers between severe or critical COVID-19 patients (designated as cases) and mild or moderate patients (designated as controls). **B** The comparison of neutralizing antibody titers between *FOXP4* rs1886814 AA + AC genotype and CC genotype. **C** The comparison of neutralizing antibody titers between *FOXP4* rs2894439 GG genotype and GA + AA genotype. **D** The comparison of neutralizing antibody titers between *MX1* rs17000900 CC genotype and CA + AA genotype in males

We examined antibody responses in COVID-19 patients and found that severe cases exhibited higher microneutralization antibody levels. For AA + AC genotype carriers of *FOXP4* rs1886814, their neutralizing antibody titers were significantly lower than that of subjects with CC genotype (*P* = 0.0004). Compared with FOXP4 rs2894439 GG carriers, GA + AA carriers had higher neutralizing antibody titers (*P* = 0.0018). The CC genotype of MX1 rs17000900 tended to be correlated with higher neutralizing antibody titers than CA + AA genotype (*P* = 0.0663), but the difference was not statistically significant (Fig. 1 and Supplementary Table 5).

### LD and haplotype analysis

The LD structures of two SNPs in *MX1* and *FOXP4* locus were presented in Supplementary Tables 6–7. Compared with the CG haplotype, the AT haplotype comprised of *MX1* rs17000900-rs2071430 was associated with protection from severe COVID-19 (*P* = 6.3E-03, OR = 0.21, 95%CI = 0.07–0.64) (Table 5). ﻿Compared with the AG haplotype, the CA haplotype composed of rs1886814-rs2894439 was associated with a higher risk of severe or critical COVID-19 (*P* = 7.0E-04, OR = 2.37, 95% CI = 1.44–3.88) (Table 6). We also explored protective haplotypes comprised of four *OAS1* and *OAS3* variants that were inherited from Neandertals [10], including *OAS1* splicing variant rs10774671, missense variants rs1131454 (Gly162Ser), rs2660 (3’UTR) and a common *OAS3*-exon 6 rs2285933 (Ser381Arg), which associated with COVID-19 severity in patients of European ancestry. We found that no haplotype was correlated with severe disease in Supplementary Tables 8–9.

**Table 5:** Association between *MX1* haplotypes and the severity of COVID-19

Haplotype		Frequency			Adjusted OR(95%CI)	*P*
rs17000900	rs2071430	Total	Case	Control		
C	G	0.71	0.75	0.70	1.00	-
C	T	0.15	0.17	0.15	1.19(0.67–2.12)	0.56
A	T	0.11	0.03	0.14	0.21(0.07–0.64)	6.3E-03
A	G	0.02	0.04	0.02	0.65(0.23–1.85)	0.42

*SNP* single nucleotide polymorphism, *OR* odds ratio, *CI* confidence interval

**Table 6:** Association between *FOXP4* haplotypes and the severity of COVID-19

Haplotype		Frequency			Adjusted OR(95%CI)	*P*
rs1886814	rs2894439	Total	Case	Control		
A	G	0.52	0.36	0.57	1.00	-
C	A	0.38	0.50	0.35	2.37(1.44–3.88)	7.0E-04
A	A	0.05	0.08	0.04	2.17(0.79–5.99)	0.14
C	G	0.05	0.08	0.04	2.52(0.87–7.26)	0.09

*SNP* single nucleotide polymorphism, *OR* odds ratio, *CI* confidence interval

## Discussion

The clinical manifestations of COVID-19 showed apparent heterogeneity, indicating that genetic factors may affect disease severity. In our study, we found that the *MX1* rs17000900 CA + AA genotype tended to be correlated with a reduced risk of severe COVID-19 than the CC genotype in males. The AT haplotype consisting of MX1 rs17000900 and rs2071430 was likely to protect against COVID-19 adverse outcomes. Moreover, we observed that rs1886814 and rs2894439 variants of *FOXP4* were associated with increased disease severity following SARS-CoV-2 infection. The CA haplotype comprised of risk alleles rs1886814 and rs2894439 was found to have a more significant association with worse clinical outcomes.

The IFN-stimulated gene MX1 encodes myxovirus resistance protein A (MxA), which belongs to the dynamin-like GTPase family and shows broad antiviral activity [11]. Previous studies have reported that MX1 polymorphisms could influence the antiviral and enzymatic activities, which can be expected to insight into the molecular mechanisms of inter-individual variabilities in susceptibility and severity of viral diseases [12]. In addition, a whole-genome sequencing study investigated the association between H7N9 infection and single-nucleotide variants in MX1, and observed that multiple MX1 rare variants increased susceptibility to the H7N9 influenza virus [13]. Among the validated variants in MX1, two promoter single-nucleotide polymorphisms (-123C > A, rs17000900; -88G > T, rs2071430) near the IFN-stimulated response element (ISRE) have been frequently reported the association with various viral diseases, including hepatitis C virus(HCV) [14], enterovirus 71(EV71) [15] and SARS-CoV [16–18]. Furthermore, higher *MX1* expression was associated with a better response to the influenza A H1N1 pandemic in 2009 [19]. MX1 was also a critical responder in SARS-CoV-2 infection. The MX1 expression level was higher in COVID-19 patients, and its expression reduced significantly with age [20], which supported increasing severity in older patients.

*MX1* rs17000900 (-123C > A) is a promoter variant near the IFN-stimulated response element. A luciferase reporter assay demonstrated that the rs17000900 A allele contributed to increased promoter activity [14]. Zhang et al. [15]reported that the rs17000900 CA + AA genotype increased expression levels than the CC genotype. The -123A allele also provided stronger binding affinity to nuclear proteins than the wild-type allele [18]. Altogether, the rs17000900 A allele plays a more critical role in the regulation of *MX1* antiviral response and then reducing the risk of disease severity. As previous studies reported [21], another five SNPs at the TMPRSS2/MX1 locus were correlated with a reduced risk of developing severe COVID-19 with the high level of MX1 expression in blood.

FOXP4 encodes a transcription factor associated with neurodevelopmental disorders and lung cancer [22]. Tian et al. [23] reported that FOXP4 was an important regulator of non-small cell lung cancer (NSCLC) and was significantly highly expressed in NSCLC cell lines and NSCLC patients. FOXP4 played a crucial role in regulating lung secretory epithelial cell fate and regeneration during lung development, and FOXP4 downregulation can impair epithelium regeneration in lung tissue [24, 25]. Thus, FOXP4 could protect the lung against pathogens by affecting the production of mucus. Furthermore, FOXP4 was also expressed in CD4 + and CD8 + T cells and necessary for memory T-cell cytokines recall responses to viral infection [26]. For COVID-19, SARS-CoV-2 cross-reactive T-cell immunity due to an exposure history to common cold coronaviruses [27] may affect disease severity.

The variant rs1886814 is located within *FOXP4-AS1*, a lncRNA gene that upregulates FOXP4 [28]. In the meantime, a recent colocalization analysis reported that this lung-specific expression quantitative trait loci (eQTL) signal for *FOXP4* identified rs1886814 as the variant with the highest likelihood of causality, which is located ∼10 kb upstream of *FOXP4* and is likely a regulatory variant associated with increased *FOXP4* expression when A allele mutates to C [29]. The GWAS meta-analysis of COVID-19 Host Genetics Initiative (HGI) demonstrated that rs1886814 was associated with the severity of COVID-19 manifestation, which was increased odds of more severe COVID-19 phenotypes [5]. Ani Manichaikul et al. [30] conducted a GWAS of subclinical interstitial lung disease (ILD) in the population-based Multi-Ethnic Study of Atherosclerosis Study, which reported a novel *FOXP4* region variant rs2894439 associated with emphysema and in LD with the lead variants rs1886814 (r^2^ = 0.7) [29], suggesting a potential role of this gene in disease severity. These data suggested the minor, expression-increasing allele is associated with an increased risk of COVID-19 disease severity and interstitial lung disease. Interestingly, the risk alleles of these two variants are much more common in East Asians than in other 1 KG populations (rs1886814 MAF = 0.381 in East Asians and 0.043 in 1KGP Europeans, rs2894439 MAF = 0.430 in East Asians and 0.031 in 1KGP Europeans). Therefore, we hypothesized that rs1886814 A allele to C change and rs2894439 G to A change may regulate *FOXP4* gene and protein expression, and then affect the production of mucus or memory T-cell recall responses to SARS-CoV-2. Wu et al. [31] identified a significant intronic variant in *FOXP4* locus associated with severe COVID-19 (rs1853837, OR = 1.28, *p* = 2.51E-10, LD r^2^ = 0.64 with rs1886814), supporting the validity of our result. The CA haplotype composed of rs1886814 and rs2894439 was found to have a more significant association with worse clinical outcomes, which was consistent with the single SNP analysis. This result further suggested that rs1886814 and rs2894439 played an important role in the severity of COVID-19.

Previous studies reported that virus-neutralizing antibodies were associated with COVID-19 severity [32, 33]. One possibility is that severe disease caused by hyperinflammation or uncontrolled viral replication induces overproduction of antibodies that serve as a “biomarker” of severity [33]. Thus, consistent with previous studies, our result suggests severity of SARS-CoV-2 infection significantly correlates with higher antibody levels.﻿ Interestingly, we observed a significant association between genotypes of rs1886814 and rs2894439 at the FOXP4 locus and the serum level of neutralizing antibodies. ﻿Further investigations are needed to understand how the FOXP4 gene affects antibody response and disease severity.

No haplotype of the OAS gene cluster was found to have an association with severe disease in our study. Related studies from Zhou [34] found that the protective alleles at both rs4767027-T (the *OAS1* pQTL) and rs10774671-G (the *OAS1* sQTL) are found on a Neanderthal haplotype. Banday [35] found that rs10774671-A and rs1131454-A formed a common haplotype, which decreased *OAS1* expression and contributed to COVID-19 severity through allele-specific regulation of splicing and nonsense-mediated decay in European and African populations. The human genetic background influences the susceptibility to and the severity of infectious diseases. Further studies of larger samples in the Asian population should be carried out.

Sex-specific differences in clinical outcomes and immune response to SARS-CoV-2 were observed in our study. This is, at least in part, due to sex-based differences in innate and adaptive immune responses that are influenced by sex-related genes and sex hormones [36]. Sex hormones regulate immune-related gene expression by binding to the receptors expressed on cells of the immune system. Notably, estrogens can promote beneficial immune system activation, which may protect against severe COVID-19, but the effects of androgens on immune function are largely suppressive [37]. The X chromosome contains many immune-related genes, and incomplete X-inactivation can provide another immune advantage to females [38].

There are still certain limitations to our study. The sample size of subjects was relatively small, thus providing only limited power. However, both the case group and the control group in this study met the minimum sample size required for the significant sites at the level of statistical efficacy of 0.80. Meanwhile, the incidence rate of the outcome event was 20.38% in our study, which was observed much the same as in previous studies [39], a report of 72,314 cases in China with 14% severe cases and 5% critical cases. We believe the association could be revealed even with a modest sample size by the candidate gene approach. The Bonferroni correction was made considering the number of SNPs analyzed. That a more conservative analysis considering the number of tests carried out could lead to a type II error, considering the sample size. In addition, these observations were consistent with large genomic studies in diverse populations by HGI [5], as this is a replication study among the Guangdong population. Finally, the specific mechanism of how the variants regulate our immune system and thus contribute to diverse clinical presentations of COVID-19 remains unknown. Therefore, further studies with large participants are required to investigate such associations and explore potential mechanisms of how SNPs affect this.

In conclusion, our study found that the *MX1* gene promoter variant tended to be associated with a reduced risk of developing severe COVID-19 in males, and the polymorphisms near *FOXP4* were significantly associated with increased COVID-19 severity in the Guangdong population. Distinguishing high-risk patients who develop severe COVID-19 will provide information for early intervention and individual treatment strategies.

### Supplementary Information

**Additional file 1: Supplementary Table 1.** The detail information of 22 Candidate gene SNPs. **Supplementary Table 2.** Genotype and allele distributions of candidate SNPs between the two groups. **Supplementary Table 3.** Multivariable logistic regression analysis adjusted for age, gender and comorbidities. **Supplementary Table 4.** Multivariable logistic regression analysis of 20 candidate variants in different gender groups. **Supplementary Table 5.** Analysis between MX1 rs17000900, FOXP4 rs1886814 and rs2894439 genotypes and neutralizing antibody titers. **Supplementary Table 6.** The linkage disequilibrium coefficients among two SNPs of MX1. **Supplementary Table 7.** The linkage disequilibrium coefficients among two SNPs of FOXP4. **Supplementary Table 8.** The linkage disequilibrium coefficients among four SNPs of OAS gene cluster. **Supplementary Table 9.** Association between haplotypes of the OAS gene cluster and the severity of COVID-19. **Supplementary Table 10.** Calculation of minimum sample size in case and control group.

## Data Availability

The datasets generated or analyzed during this study are not publicly available due to existing general data protection rules and official secrecy. However, it could be available from the corresponding author on reasonable request.
