# Supplementary material for: Polymorphisms of IFN signaling genes and FOXP4 influence the severity of COVID-19
Source: BMC Infect Dis. 2024 Mar 1;24:270. doi: 10.1186/s12879-024-09040-6 (PMC10905836; doi:10.1186/s12879-024-09040-6)
Supplement: Supplementary file 1 — Additional file 1: Supplementary Table 1. The detail information of 22 Candidate gene SNPs. Supplementary Table 2. Genotype and allele distributions of candidate SNPs between the two groups. Supplementary Table 3. Multivariable logistic regression analysis adjusted for age, gender and comorbidities. Supplementary Table 4. Multivariable logistic regression analysis of 20 candidate variants in different gender groups. Supplementary Table 5. Analysis between MX1 rs17000900, FOXP4 rs1886814 and rs2894439 genotypes and neutralizing antibody titers. Supplementary Table 6. The linkage disequilibrium coefficients among two SNPs of MX1. Supplementary Table 7. The linkage disequilibrium coefficients among two SNPs of FOXP4. Supplementary Table 8. The linkage disequilibrium coefficients among four SNPs of OAS gene cluster. Supplementary Table 9. Association between haplotypes of the OAS gene cluster and the severity of COVID-19. Supplementary Table 10. Calculation of minimum sample size in case and control group. [file 12879_2024_9040_MOESM1_ESM.docx]

**Polymorphisms of *IFN* signaling genes and *FOXP4* influence the severity of COVID-19**

| **Supplementary Table 1.** The detail information of 22 Candidate gene SNPs. | | | | | | | | |
| --- | --- | --- | --- | --- | --- | --- | --- | --- |
| **Gene** | **Chromosome**  **location** | **SNP** | **Function** | **Allele** | **CHB MAF** | **MAF** | **Call rates (%)** | ***P*_HWE_^a^** |
| *TLR3* | 4:186082920 | rs3775291 | missense | C/T | 0.291 | 0.232 | 100 | 0.920 |
|  | 4:186079213 | rs5743313 | intron | C/T | 0.136 | 0.186 | 100 | 0.845 |
| *TLR7* | X:12889539 | rs3853839 | 3' UTR | G/C | 0.237 | 0.402 | 100 | **0.000** |
| *DDX58* | 9:32526148 | rs10813831 | missense | G/A | 0.083 | 0.184 | 100 | 0.102 |
|  | 9:32526235 | rs3739674 | intron | G/C | 0.456 | 0.463 | 100 | 0.239 |
| *IFIH1* | 2:162267541 | rs1990760 | missense | C/T | 0.146 | 0.357 | 100 | 0.483 |
|  | 2:162254026 | rs2111485 | regulatory region variant | A/G | 0.141 | 0.339 | 100 | 0.906 |
| *IFNAR2* | 21:33252612 | rs2236757 | intron | A/G | 0.383 | 0.375 | 100 | 0.273 |
|  | 21:33242905 | rs13050728 | intron | T/C | 0.383 | 0.398 | 100 | **0.000** |
|  | 21:33241950 | rs1051393 | missense | G/T | 0.383 | 0.392 | 100 | 0.378 |
|  | 21:33241945 | rs2229207 | missense | T/C | 0.214 | 0.119 | 100 | 0.179 |
| *TYK2* | 19:10364976 | rs2304256 | missense | C/A | 0.456 | 0.266 | 100 | 0.873 |
| *MX1* | 21:41426103 | rs17000900 | 5'UTR | C/A | 0.175 | 0.172 | 100 | 0.645 |
|  | 21:41426138 | rs2071430 | 5' UTR | G/T | 0.325 | 0.238 | 100 | 0.008 |
| *OAS1* | 12:112919388 | rs10774671 | splice acceptor variant | A/G | 0.306 | 0.386 | 100 | 0.032 |
|  | 12:112911065 | rs1131454 | missense | A/G | 0.471 | 0.474 | 100 | 0.984 |
|  | 12:112919637 | rs2660 | stop gained | A/G | 0.306 | 0.212 | 100 | 0.342 |
| *OAS3* | 12:112942203 | rs10735079 | 3' UTR | A/G | 0.306 | 0.263 | 100 | 0.530 |
|  | 12:112948974 | rs2285933 | missense | C/G | 0.155 | 0.281 | 100 | 0.735 |
|  | 12:112938583 | rs1859330 | missense | A/G | 0.301 | 0.337 | 98 | 0.171 |
| *FOXP4* | 6:41534945 | rs1886814 | intron | A/C | 0.364 | 0.157 | 100 | 0.966 |
|  | 6:41512355 | rs2894439 | intron | G/A | 0.388 | 0.163 | 100 | 0.516 |
| ^a^ *P_HWE_* was the value of Hardy-Weinberg equilibrium test. | | | | | | | | |

| **Supplementary Table 2.** Genotype and allele distributions of candidate SNPs between the two groups. | | | | | | | |
| --- | --- | --- | --- | --- | --- | --- | --- |
| **Gene** | **SNP** | **Genotype** | **Case(n=64)** | **Control(n=250)** | ***P*^a^** | **OR(95%CI)^b^** | ***P*^b^** |
| *TLR3* | rs3775291 | CC | 29(45.3) | 102(40.8) | 0.798 | 1.000 |  |
|  |  | CT | 27(42.2) | 116(46.4) |  | 0.819(0.455-1.474) | 0.505 |
|  |  | TT | 8(12.5) | 32(12.8) |  | 0.879(0.366-2.115) | 0.774 |
|  |  | C | 85(66.4) | 320(64.0) | 0.612 |  |  |
|  |  | T | 43(33.6) | 180(36.0) |  |  |  |
|  | rs5743313 | CC | 46(71.9) | 198(79.2) | 0.349**^c^** | 1.000 |  |
|  |  | CT | 17(26.6) | 49(19.6) |  | 1.493(0.789-2.827) | 0.218 |
|  |  | TT | 1(1.6) | 3(1.2) |  | 1.435(0.146-14.109) | 0.757 |
|  |  | C | 109(85.2) | 445(89.0) | 0.229 |  |  |
|  |  | T | 19(14.8) | 55(11.0) |  |  |  |
| *DDX58* | rs10813831 | GG | 52(81.3) | 192(76.8) | 0.704 | 1.000 |  |
|  |  | GA | 11(17.2) | 51(20.4) |  | 0.796(0.388-1.636) | 0.535 |
|  |  | AA | 1(1.6) | 7(2.8) |  | 0.527(0.063-4.384) | 0.554 |
|  |  | G | 115(89.8) | 435(87.0) | 0.384 |  |  |
|  |  | A | 13(10.2) | 65(13.0) |  |  |  |
|  | rs3739674 | GG | 23(35.9) | 87(34.8) | 0.981 | 1.000 |  |
|  |  | GC | 29(45.3) | 114(45.6) |  | 0.962(0.521-1.779) | 0.902 |
|  |  | CC | 12(18.8) | 49(19.6) |  | 0.926(0.4242-2.02) | 0.848 |
|  |  | G | 75(58.6) | 288(57.6) | 0.839 |  |  |
|  |  | C | 53(41.4) | 212(42.4) |  |  |  |
| *IFIH1* | rs1990760 | CC | 41(67.2) | 170(68.0) | 0.108 | 1.000 |  |
|  |  | CT | 19(29.7) | 76(30.4) |  | 1.037(0.565-1.903) | 0.908 |
|  |  | TT | 4(6.3) | 4(1.6) |  | 4.146(0.995-17.279) | 0.051 |
|  |  | C | 101(78.9) | 416(83.2) | 0.256 |  |  |
|  |  | T | 27(21.1) | 84(16.8) |  |  |  |
|  | rs2111485 | AA | 41(64.1) | 179(71.6) | 0.317 | 1.000 |  |
|  |  | AG | 20(31.3) | 66(26.4) |  | 1.323(0.723-2.422) | 0.364 |
|  |  | GG | 3(4.7) | 5(2.0) |  | 2.620(0.602-11.405) | 0.199 |
|  |  | A | 102(79.7) | 424(84.8) | 0.162 |  |  |
|  |  | G | 26(20.3) | 76(15.2) |  |  |  |
| *IFNAR2* | rs2236757 | AA | 27(42.2) | 103(41.2) | 0.972 | 1.000 |  |
|  |  | AG | 28(43.8) | 109(43.6) |  | 0.980(0.541-1.774) | 0.947 |
|  |  | GG | 9(14.1) | 38(15.2) |  | 0.904(0.390-2.095) | 0.813 |
|  |  | A | 82(64.1) | 315(63.0) | 0.824 |  |  |
|  |  | G | 46(35.9) | 185(37.0) |  |  |  |
|  | rs1051393 | GG | 28(43.8) | 103(41.2) | 0.932 | 1.000 |  |
|  |  | GT | 27(42.2) | 111(44.4) |  | 0.895(0.495-1.619) | 0.713 |
|  |  | TT | 9(14.1) | 36(14.4) |  | 0.920(0.396-2.133) | 0.845 |
|  |  | G | 83(64.8) | 317(63.4) | 0.762 |  |  |
|  |  | T | 45(35.2) | 183(36.6) |  |  |  |
|  | rs2229207 | TT | 39(60.9) | 157(62.8) | 0.710 | 1.000 |  |
|  |  | TC | 24(37.5) | 85(34.0) |  | 1.137(0.641-2.016) | 0.661 |
|  |  | CC | 1(1.6) | 8(3.2) |  | 0.503(0.061-4.143) | 0.523 |
|  |  | T | 102(79.7) | 399(79.8) | 0.977 |  |  |
|  |  | C | 26(20.3) | 101(20.2) |  |  |  |
| *TYK2* | rs2304256 | CC | 16(25.0) | 54(21.4) | 0.664 | 1.00 |  |
|  |  | CA | 29(45.3) | 129(51.6) |  | 0.76(0.38-1.51) | 0.432 |
|  |  | AA | 19(29.7) | 67(26.8) |  | 0.96(0.45-2.04) | 0.909 |
|  |  | C | 61(47.7) | 237(47.4) | 0.959 |  |  |
|  |  | A | 67(52.3) | 263(52.6) |  |  |  |
| *MX1* | rs17000900 | CC | 54(84.4) | 180(72.0) | 0.089 | 1.000 |  |
|  |  | CA | 10(15.6) | 63(25.2) |  | 0.529(0.254-1.101) | 0.089 |
|  |  | AA | 0(0.0) | 7(2.8) |  | - | - |
|  |  | C | 118(92.2) | 423(84.6) | 0.027 |  |  |
|  |  | A | 10(7.8) | 77(15.4) |  |  |  |
|  | rs2071430 | GG | 42(65.6) | 135(54.0) | 0.210 | 1.000 |  |
|  |  | GT | 18(28.1) | 87(34.8) |  | 0.665(0.360-1.229) | 0.193 |
|  |  | TT | 4(6.3) | 28(11.2) |  | 0.459(0.152-1.384) | 0.167 |
|  |  | G | 102(79.7) | 357(71.3) | 0.059 |  |  |
|  |  | T | 26(20.3) | 143(28.6) |  |  |  |
| *OAS1* | rs10774671 | AA | 36(56.3) | 155(62.0) | 0.679 | 1.000 |  |
|  |  | AG | 23(35.9) | 76(30.4) |  | 1.303(0.722-2.352) | 0.380 |
|  |  | GG | 5(7.8) | 19(7.6) |  | 1.133(0.397-3.237) | 0.816 |
|  |  | A | 95(74.2) | 386(77.2) | 0.477 |  |  |
|  |  | G | 33(25.8) | 114(22.8) |  |  |  |
|  | rs1131454 | AA | 22(34.4) | 83(33.2) | 0.944 | 1.000 |  |
|  |  | AG | 30(46.9) | 123(49.2) |  | 0.920(0.497-1.705) | 0.791 |
|  |  | GG | 12(18.8) | 44(17.6) |  | 1.029(0.466-2.273) | 0.944 |
|  |  | A | 74(57.8) | 289(57.8) | 0.998 |  |  |
|  |  | G | 54(42.2) | 211(42.2) |  |  |  |
|  | rs2660 | AA | 36(56.3) | 155(62.0) | 0.643 | 1.000 |  |
|  |  | AG | 23(35.9) | 81(32.4) |  | 1.223(0.679-2.201) | 0.503 |
|  |  | GG | 5(7.8) | 14(5.6) |  | 1.538(0.520-4.544) | 0.436 |
|  |  | A | 95(74.2) | 391(77.4) | 0.337 |  |  |
|  |  | G | 33(25.8) | 109(21.8) |  |  |  |
| *OAS3* | rs10735079 | AA | 36(56.3) | 154(61.6) | 0.736 | 1.000 |  |
|  |  | AG | 24(37.5) | 82(32.8) |  | 1.25(0.70-2.24) | 0.449 |
|  |  | GG | 4(6.3) | 14(5.6) |  | 1.22(0.38-3.93) | 0.737 |
|  |  | A | 96(75.0) | 390(78.0) | 0.469 |  |  |
|  |  | G | 32(25.0) | 110(22.0) |  |  |  |
|  | rs2285933 | CC | 49(76.6) | 159(63.6) | 0.090 | 1.000 |  |
|  |  | CG | 12(18.8) | 82(32.8) |  | 0.475(0.239-0.942) | 0.033 |
|  |  | GG | 3(4.7) | 9(3.6) |  | 1.082(0.282-4.153) | 0.909 |
|  |  | C | 110(85.9) | 400(80.0) | 0.125 |  |  |
|  |  | G | 18(14.1) | 100(20.0) |  |  |  |
|  | rs1859330 | AA | 36(57.1) | 158(64.8) | 0.526 | 1.000 |  |
|  |  | AG | 23(36.5) | 72(29.5) |  | 1.402(0.775-2.536) | 0.264 |
|  |  | GG | 4(6.3) | 14(5.7) |  | 1.254(0.390-4.035) | 0.704 |
|  |  | A | 95(75.4) | 388(79.5) | 0.315 |  |  |
|  |  | G | 31(24.6) | 100(20.5) |  |  |  |
| *FOXP4* | rs1886814 | AA | 13(20.3) | 90(36.0) | **3.89E-04** | 1.000 |  |
|  |  | AC | 29(45.3) | 125(50.0) |  | 1.606(0.791-3.261) | 0.190 |
|  |  | CC | 22(34.4) | 35(14.0) |  | 4.352(1.977-9.578) | 2.59E-04 |
|  |  | A | 55(43.0) | 305(61.0) | **2.33E-04** |  |  |
|  |  | C | 73(57.0) | 195(39.0) |  |  |  |
|  | rs2894439 | GG | 10(15.6) | 96(38.4) | **0.001** | 1.000 |  |
|  |  | GA | 35(54.7) | 113(45.2) |  | 2.973(1.400-6.317) | 0.005 |
|  |  | AA | 19(29.7) | 41(16.4) |  | 4.449(1.904-10.393) | 0.001 |
|  |  | G | 55(43.0) | 305(61.0) | **2.33E-04** |  |  |
|  |  | A | 73(57.0) | 195(39.0) |  |  |  |
| ﻿^a^ The *P* values were calculated by the Chi-square test. ^b^ ORs and *P* values were calculated by univariable logistic  regression analysis. ^c^Fisher's exact probability test. | | | | | | | |

| **Supplementary Table 3.** Multivariable logistic regression analysis adjusted for age, gender and comorbidities. | | | | | | | |
| --- | --- | --- | --- | --- | --- | --- | --- |
| **Gene** | **SNP** | **Genetic model** | **Genotype** | **Case** | **Control** | ***P*** | **OR(95%CI)** |
| *TLR3* | rs3775291 | Codominant | CC | 28(45.9) | 92(39.8) |  | 1.000 |
|  |  |  | CT | 25(41.0) | 108(46.8) | 0.033 | 0.439(0.206-0.936) |
|  |  |  | TT | 8(13.1) | 31(13.4) | 0.493 | 0.686(0.234-2.014) |
|  | rs5743313 | Overdominant | CC+TT | 44(72.1) | 189(81.8) |  | 1.000 |
|  |  |  | CT | 17(27.9) | 42(18.2) | 0.017 | 2.585(1.185-5.641) |
| *DDX58* | rs10813831 | Recessive | GG+GA | 60(98.4) | 226(97.8) |  | 1.000 |
|  |  |  | AA | 1(1.6) | 5(2.2) | 0.676 | 0.584(0.047-7.286) |
|  | rs3739674 | Codominant | GG | 21(34.4)) | 80(34.6) |  | 1.000 |
|  |  |  | GC | 28(45.9) | 106(45.9) | 0.698 | 1.166(0.537-2.529) |
|  |  |  | CC | 12(19.7) | 45(19.5) | 0.404 | 1.488(0.585-3.785) |
| *IFIH1* | rs1990760 | Codominant | CC | 40(65.6) | 158(63.4) |  | 1.000 |
|  |  |  | CT | 18(29.5) | 70(30.3) | 0.635 | 1.194(0.574-2.482) |
|  |  |  | TT | 3(4.9) | 3(1.3) | 0.125 | 4.157(0.672-25.710) |
|  | rs2111485 | Dominant | GG | 40(65.6) | 166(71.9) |  | 1.000 |
|  |  |  | GC+CC | 21(34.4) | 65(28.1) | 0.287 | 1.467(0.724-2.969) |
| *IFNAR2* | rs2236757 | Dominant | AA | 26(42.6) | 96(41.6) |  | 1.000 |
|  |  |  | AG+GG | 35(57.4) | 135(58.4) | 0.326 | 0.711(0.360-1.405) |
|  | rs1051393 | Dominant | GG | 27(44.3) | 96(41.6) |  | 1.000 |
|  |  |  | GT+TT | 34(55.7) | 135(58.4) | 0.257 | 0.675(0.341-1.333) |
|  | rs2229207 | Recessive | TT+TC | 60(98.4) | 223(96.5) |  | 1.00 |
|  |  |  | CC | 1(1.6) | 8(3.5) | 0.741 | 0.686(0.073-6.425) |
| *TYK2* | rs2304256 | Dominant | CC | 13(21.3) | 51(22.1) |  | 1.000 |
|  |  |  | CA+AA | 48(78.7) | 180(77.9) | 0.852 | 1.080(0.481-2.422) |
| *MX1* | rs17000900 | Dominant | CC | 52(85.2) | 165(71.4) |  | 1.000 |
|  |  |  | CA+AA | 9(14.8) | 66(28.6) | 0.013 | 0.313(0.125-0.787) |
|  | rs2071430 | Dominant | GG | 41(67.2) | 125(54.1) |  | 1.000 |
|  |  |  | GT+TT | 20(32.8) | 106(45.9) | 0.364 | 0.728(0.367-1.444) |
| *OAS1* | rs10774671 | Recessive | AA+AG | 58(95.1) | 215(93.1) |  | 1.000 |
|  |  |  | GG | 3(4.9) | 16(6.9) | 0.576 | 0.658(0.152-2.849) |
|  | rs1131454 | Overdominant | AA+GG | 32(52.5) | 116(50.2) |  | 1.000 |
|  |  |  | AG | 29(47.5) | 115(49.8) | 0.280 | 0.686(0.346-1.359) |
|  | rs2660 | Codominant | AA | 36(59.0) | 140(60.6) |  | 1.000 |
|  |  |  | AG | 22(36.1) | 79(34.2) | 0.942 | 1.027(0.506-2.082) |
|  |  |  | GG | 3(4.9) | 12(5.2) | 0.726 | 1.293(0.308-5.425) |
| *OAS3* | rs10735079 | Recessive | AA+AG | 59(96.7) | 219(94.8) |  | 1.000 |
|  |  |  | GG | 2(3.3) | 12(5.2) | 0.868 | 0.871(1.71-4.436) |
|  | rs2285933 | Overdominant | CC+GG | 49(80.3) | 156(67.5) |  | 1.000 |
|  |  |  | CG | 12(19.7) | 75(32.5) | 0.043 | 0.426(0.186-0.975) |
|  | rs1859330 | Overdominant | AA+GG | 58(96.7) | 213(94.7) |  | 1.000 |
|  |  |  | AG | 2(3.3) | 12(5.3) | 0.321 | 1.423(0.709-2.859) |
| *FOXP4* | rs1886814 | Recessive | AA+AC | 39(63.9) | 200(86.6) |  | 1.000 |
|  |  |  | CC | 22(36.1) | 31(13.4) | 0.001 | 3.747(1.746-8.043) |
|  | rs2894439 | Dominant | GG | 7(11.5) | 88(38.1) |  | 1.000 |
|  |  |  | GA+AA | 54(88.5) | 143(61.9) | 0.001 | 5.703(2.045-15.903) |
| ^a^ ORs and *P* values were calculated by multivariable logistic regression analysis adjusted for age, gender and comorbidities. | | | | | | | |

| **Supplementary Table 4**. Multivariable logistic regression analysis of 20 candidate variants in different gender groups | | | | | | | | | | | | |
| --- | --- | --- | --- | --- | --- | --- | --- | --- | --- | --- | --- | --- |
| Gene | SNP | Genetic Model | Genotype | Male | | | |  | Female | | | |
|  |  |  |  | Case | Control | *P* | OR(95%CI) |  | Case | Control | *P* | OR(95%CI) |
| *TLR3* | rs3775291 | Codominant | CC | 15(37.5) | 41(39.8) |  | 1.000 |  | 13(61.9) | 51(39.8) |  | 1.000 |
|  |  |  | CT | 19(47.5) | 49(47.6) | 0.406 | 0.603(0.229-1.588) |  | 6(28.6) | 59(46.1) | 0.033 | 0.252(0.071-0.892) |
|  |  |  | TT | 6(15.0) | 13(12.6) | 0.759 | 1.236(0.319-4.759) |  | 2(9.5) | 18(14.1) | 0.127 | 0.181(0.020-1.625) |
|  | rs5743313 | Overdominant | CC+TT | 30(75.0) | 84(81.6) |  | 1.000 |  | 14(66.7) | 105(82.0) |  | 1.000 |
|  |  |  | CT | 10(25.0) | 19(18.4) | 0.270 | 1.771(0.642-4.888) |  | 7(33.3) | 23(18.0) | 0.016 | 4.711(1.341-16.549) |
| *DDX58* | rs10813831 | Recessive | GG+GA | 40(100.0) | 100(97.1) |  | 1.000 |  | 20(95.2) | 126(98.4) |  | 1.000 |
|  |  |  | AA | 0(0.0) | 3(2.9) | - | - |  | 1(4.8) | 2(1.6) | 0.994 | 0.987(0.041-23.519) |
|  | rs3739674 | Codominant | GG | 14(35.0) | 39(37.9) |  | 1.000 |  | 7(33.3) | 41(32.0) |  | 1.000 |
|  |  |  | GC | 18(45.0) | 42(40.8) | 0.289 | 1.750(0.622-4.921) |  | 10(47.6) | 64(50.0) | 0.419 | 0.601(0.174-2.070) |
|  |  |  | CC | 8(20.0) | 22(21.3) | 0.247 | 2.076(0.602-7.151) |  | 4(19.1) | 23(18.0) | 0.789 | 0.808(0.170-3.851) |
| *IFIH1* | rs1990760 | Codominant | CC | 29(72.5) | 73(70.9) |  | 1.000 |  | 11(52.4) | 85(66.4) |  | 1.000 |
|  |  |  | CT | 9(22.5) | 28(27.2) | 0.888 | 0.931(0.342-2.532) |  | 9(42.8) | 42(32.8) | 0.366 | 1.664(0.552-5.012) |
|  |  |  | TT | 2(5.0) | 2(1.9) | 0.304 | 3.452(0.326-3.596) |  | 1(4.8) | 1(0.8) | 0.223 | 6.397(0.323-12.668) |
|  | rs2111485 | Dominant | AA | 29(72.5) | 75(72.8) |  | 1.000 |  | 11(52.4) | 91(71.1) |  | 1.000 |
|  |  |  | AG+GG | 11(27.5) | 28(27.2) | 0.787 | 1.140(0.439-2.958) |  | 10(47.6) | 37(28.9) | 0.178 | 2.104(0.713-6.211) |
| *IFNAR2* | rs2236757 | Dominant | AA | 20(50.0) | 43(41.7) |  | 1.000 |  | 6(28.6) | 53(41.4) |  | 1.000 |
|  |  |  | AG+GG | 20(50.0) | 60(58.3) | 0.061 | 0.412(0.164-1.040) |  | 15(71.4) | 75(58.6) | 0.399 | 1.631(0.524-5.076) |
|  | rs1051393 | Dominant | GG | 20(50.0) | 44(42.7) |  | 1.000 |  | 7(33.3) | 52(40.6) |  | 1.000 |
|  |  |  | GT+TT | 20(50.0) | 59(52.3) | 0.069 | 0.424(0.168-1.070) |  | 14(66.7) | 76(59.4) | 0.607 | 1.341(0.438-4.102) |
|  | rs2229207 | Recessive | TT+TC | 39(97.5) | 99(96.1) |  | 1.000 |  | 21(100.0) | 124(96.9) |  | 1.000 |
|  |  |  | CC | 1(2.5) | 4(3.9) | 0.692 | 1.580(0.164-15.211) | | 0(0.0) | 4(3.1) | - | - |
| *TYK2* | rs2304256 | Dominant | CC | 11(27.5) | 23(22.3) |  | 1.000 |  | 2(9.5) | 28(21.9) |  | 1.000 |
|  |  |  | CA+AA | 29(72.5) | 80(77.7) | 0.359 | 0.632(0.237-1.685) |  | 19(90.5) | 100(78.1) | 0.136 | 3.596(0.668-19.371) |
| *MX1* | rs17000900 | Dominant | CC | 37(92.5) | 70(68.0) |  | 1.000 |  | 15(71.4) | 95(74.2) |  | 1.000 |
|  |  |  | CA+AA | 3(7.5) | 33(32.0) | 0.001 | 0.050(0.008-0.316) |  | 6(28.6) | 33(25.8) | 0.588 | 1.391(0.422-4.592) |
|  | rs2071430 | Dominant | GG | 29(72.5) | 53(51.5) |  | 1.000 |  | 12(57.1) | 72(56.3) |  | 1.000 |
|  |  |  | GT+TT | 11(27.5) | 50(48.5) | 0.311 | 0.628(0.255-1.545) |  | 9(42.9) | 56(43.7) | 0.826 | 0.886(0.302-2.599) |
| *OAS1* | rs10774671 | Recessive | AA+AG | 39(97.5) | 95(92.2) |  | 1.000 |  | 19(90.5) | 120(93.7)) |  | 1.000 |
|  |  |  | GG | 1(2.5) | 8(7.8) | 0.641 | 0.594(0.066-5.332) |  | 2(9.5) | 8(7.3) | 0.646 | 0.602(0.069-5.236) |
|  | rs1131454 | Overdominant | AA+GG | 23(57.5) | 50(48.5) |  | 1.000 |  | 9(42.9) | 66(51.6)) |  | 1.000 |
|  |  |  | AG | 17(42.5) | 53(51.5) | 0.232 | 0.576(0.233-1.425) |  | 12(57.1) | 62(48.4) | 0.808 | 0.873(0.294-2.597) |
|  | rs2660 | Codominant | AA | 25(62.5) | 59(57.3) |  | 1.000 |  | 11(52.4) | 81(63.3) |  | 1.000 |
|  |  |  | AG | 13(32.5) | 37(35.9) | 0.895 | 0.940(0.376-2.347) |  | 9(42.8) | 42(32.8) | 0.799 | 1.156(0.378-3.538) |
|  |  |  | GG | 2(5.0) | 7(6.8) | 0.816 | 1.237(0.207-7.406) |  | 1(4.8) | 5(3.9) | 0.923 | 1.140(0.079-16.410) |
| *OAS3* | rs10735079 | Recessive | AA+AG | 39(97.5) | 96(93.2) |  | 1.000 |  | 20(95.2) | 123(96.1) |  | 1.000 |
|  |  |  | GG | 1(2.5) | 7(6.8) | 0.726 | 0.672(0.073-6.181) |  | 1(4.8) | 5(3.9) | 0.956 | 1.076(0.078-14.924) |
|  | rs2285933 | Overdominant | CC+GG | 32(80.0) | 67(65.0) |  | 1.000 |  | 17(81.0) | 89(69.5) |  | 1.000 |
|  |  |  | CG | 8(20.0) | 36(35.0) | 0.084 | 0.403(0.144-1.131) |  | 4(19.0) | 39(30.5) | 0.267 | 0.442(0.104-1.871) |
|  | rs1859330 | Overdominant | AA+GG | 26(66.7) | 67(67.8) |  | 1.000 |  | 12(57.1) | 88(69.8) |  | 1.000 |
|  |  |  | AG | 13(33.3) | 32(32.2) | 0.589 | 1.284(0.519-3.177) |  | 9(42.9) | 38(30.2) | 0.355 | 1.681(0.559-5.058) |
| *FOXP4* | rs1886814 | Recessive | AA+AC | 26(65.0) | 89(86.4) |  | 1.000 |  | 13(61.9) | 111(86.7) |  | 1.000 |
|  |  |  | CC | 14(35.0) | 14(13.6) | 0.006 | 4.082(1.498-11.124) | | 8(38.1) | 17(13.3) | 0.037 | 3.591(1.082-11.923) |
|  | rs2894439 | Dominant | GG | 4(10.0) | 37(35.9) |  | 1.000 |  | 3(14.3) | 51(39.8) |  | 1.000 |
|  |  |  | GA+AA | 36(90.0) | 66(64.1)) | 0.018 | 4.601(1.293-16.365) | | 18(85.7) | 77(60.2) | 0.020 | 11.947(1.467-97.305) |

| **Supplementary Table 5.** Analysis between *MX1* rs17000900, *FOXP4* rs1886814 and rs2894439 genotypes and neutralizing antibody titers | | | | | |
| --- | --- | --- | --- | --- | --- |
| Gene | SNP | Genetic Model | Genotype | NAb levels ($\bar{\chi}\pm s$) | *P* |
| MX1 | rs17000900 | Dominant | CC | 1.890$\pm$0.573 | 0.173^a^ |
|  |  |  | CA+AA | 1.778$\pm$0.735 |  |
| FOXP4 | rs1886814 | Recessive | AA+AC | 1.888$\pm$0.655 | 0.012 |
|  |  |  | CC | 2.170$\pm$0.578 |  |
|  | rs2894439 | Dominant | GG | 1.763$\pm$0.660 | 0.003 |
|  |  |  | GA+AA | 2.029$\pm$0.627 |  |
| *^a^* Adjusted for age and comorbidities. *P* value for multivariate linear regression analysis adjusted for gender, age and comorbidities. | | | | | |

| **Supplementary Table 6.** The linkage disequilibrium coefficients among two SNPs of *MX1*. | | |
| --- | --- | --- |
|  | rs17000900 | rs2071430 |
| rs17000900 | - | 0.7625 |
| rs2071430 | 0.2540 | - |
| Values on the left of “-” are r^2^ and on the right are Lewontin’s D’ coefficients. | | |

| **Supplementary Table 7.** The linkage disequilibrium coefficients among two SNPs of *FOXP4*. | | |
| --- | --- | --- |
|  | rs1886814 | rs2894439 |
| rs1886814 | - | 0.8019 |
| rs2894439 | 0.6198 | - |
| Values on the left of “-” are r^2^ and on the right are Lewontin’s D’ coefficients. | | |

| **Supplementary Table 8.** The linkage disequilibrium coefficients among four SNPs of *OAS* gene cluster. | | | | |
| --- | --- | --- | --- | --- |
|  | rs10774671 | rs1131454 | rs2660 | rs2285933 |
| rs10774671 | - | 0.9141 | 0.9908 | 0.8722 |
| rs1131454 | 0.3499 | - | 0.9850 | 0.4244 |
| rs2660 | 0.9386 | 0.3884 | - | 0.8630 |
| rs2285933 | 0.0538 | 0.0571 | 0.0504 | - |
| Values on the left of “-” are r^2^ and on the right are Lewontin’s D’ coefficients. | | | | |

| **Supplementary Table 9.** Association between haplotypes of the OAS gene cluster and the severity of COVID-19. | | | | | |
| --- | --- | --- | --- | --- | --- |
| **Haplotype^a^** | **Frequency** | | | **Adjusted**  **OR(95%CI)** | ***P*** |
|  | **Total** | **Case** | **Control** |  |  |
| AAAC | 0.51 | 0.53 | 0.51 | 1.00 | - |
| GGGC | 0.22 | 0.25 | 0.21 | 0.99(0.58-1.73) | 0.72 |
| AGAG | 0.13 | 0.11 | 0.13 | 0.88(0.43-1.80) | 0.99 |
| AGAC | 0.07 | 0.06 | 0.07 | 1.01(0.16-2.00) | 0.38 |
| AAAG | 0.06 | 0.03 | 0.06 | 0.88(0.14-5.58) | 0.89 |
| Rare^b^ | - | - | - | - | - |
| ^a^ Order of SNPs for haplotype analysis is rs10774671, rs1131454, rs2660, and rs2285933. ^b^ Haplotypes with frequencies <0.01. | | | | | |

| **Supplementary Table 10**. Calculation of minimum sample size in case and control group | | | | | | | | |
| --- | --- | --- | --- | --- | --- | --- | --- | --- |
| SNPs | Genetic Model | OR | MAF | Population risk | Power | α | Case | Control |
| rs17000900 | Dominant | 0.050 | 0.175 | 64/314=0.204 | 0.80 | 0.05 | 13 | 51 |
| rs1886814 | Recessive | 3.747 | 0.364 | 64/314=0.204 | 0.80 | 0.05 | 40 | 157 |
| rs2894439 | Dominant | 5.703 | 0.388 | 64/314=0.204 | 0.80 | 0.05 | 17 | 67 |
